# Supplementary material for: Polyketide synthase gene clusters in the ericoid mycorrhizal fungus Oidiodendron maius: genome-wide prediction, expression profiling, and functional characterization of knockout mutants
Source: Front Plant Sci. 2026 Sep 16;17:1924430. doi: 10.3389/fpls.2026.1924430 (PMC13623903; doi:10.3389/fpls.2026.1924430)
Supplement: Supplementary file 1 [file SupplementaryFile1.zip › Supplementary Materials/Supplementary Folder 1.zip!/Oidma1_AssemblyScaffolds_Repeatmasked/knownclusterblast/cluster13/jgi.p_Oidma1_114547_mibig_hits.html]

| MIBiG Protein | Description | MIBiG Cluster | MiBiG Product | % ID | % Coverage | BLAST Score | E-value |
| --- | --- | --- | --- | --- | --- | --- | --- |
| ANF07284.1 | MFS | BGC0001385 | Byssochlamic\_acid\_/\_agnestadrides\_biosynthetic\_gene\_cluster | 53 | 79.8479087452 | 410 | 4.2e-114 |
| AMY15063.1 | MFS\_transporter | BGC0001339 | Squalestatin\_S1\_biosynthetic\_gene\_cluster | 41 | 88.9733840304 | 352 | 6.1e-97 |
| AMY15073.1 | MFS\_transporter | BGC0001339 | Squalestatin\_S1\_biosynthetic\_gene\_cluster | 41 | 90.1140684411 | 347 | 2.6e-95 |
| ALS30797.1 | itaconate\_transport\_protein\_1 | BGC0001286 | Itaconic\_acid\_biosynthetic\_gene\_cluster | 35 | 94.4866920152 | 258 | 2e-68 |
| BAK26557.1 | putative\_MFS\_multidrug\_transporter | BGC0000977 | Cyclopiazonic\_acid\_biosynthetic\_gene\_cluster | 26 | 87.4524714829 | 125 | 2.1e-28 |
